# Barriers and facilitators of implementing a shallow rental subsidy program at an area agency on aging

**DOI:** 10.1093/geroni/igag015

**Published:** 2026-02-13

**Authors:** Katie Hoops Calhoun, Anthony Traver, Marisa Sheldon

**Affiliations:** College of Social Work, Ohio State University, Columbus, Ohio, United States; Age-Friendly Innovation Center, Ohio State University, Columbus, Ohio, United States; Age-Friendly Innovation Center, Ohio State University, Columbus, Ohio, United States; College of Social Work, University of Kentucky, Lexington, Kentucky, United States; College of Social Work, Ohio State University, Columbus, Ohio, United States; Age-Friendly Innovation Center, Ohio State University, Columbus, Ohio, United States

**Keywords:** Area Agencies on Aging, Shallow subsidies, Implementation science

## Abstract

**Background and Objectives:**

As the number of older adults experiencing homelessness and housing insecurity grows, there is an increasing need for responses aimed at preventing housing loss in later life. Because Area Agencies on Aging (AAAs) serve older adults across the socioeconomic spectrum and all older adults in the United States have access to AAA programs that support maintaining community-based living, they are well-positioned to address housing challenges. However, little is known about the implementation of these programs, limiting the adoption of practices across the AAA network. Using the Consolidated Framework for Implementation Research (CFIR), this study aims to identify facilitators and barriers to implementing a shallow rental subsidy program at an AAA.

**Research Design and Methods:**

We used a sequential mixed-methods implementation science design. Quantitative data were collected from AAA staff through a survey, and qualitative data were collected through focus groups.

**Results:**

Barriers and facilitators were identified across the 11 constructs within the CFIR inner-setting domain. Facilitators to implementation included the AAA’s client-centered culture and structural characteristics (e.g., having an established Housing Assistance Program). Barriers to implementation included incompatibility with case manager workloads and a short timeframe that created confusion about the program.

**Discussion and Implications:**

Findings highlight opportunities for AAAs to implement shallow rental subsidy programs. Having an existing housing team structure is a facilitator for implementing new housing-related programs (e.g., shallow subsidies). Additionally, clear, targeted communication about the rationale for program elements and adequate time for referral, screening, and enrollment are essential for successful implementation.

Innovation and Translational Significance: Given the rising number of older adults experiencing housing instability in the United States, this manuscript describes how one Area Agency on Aging (AAA) implemented an innovative rental assistance program to support older adults. Using the Consolidated Framework for Implementation Research (CFIR), this article draws on qualitative and quantitative data from surveys and focus groups to highlight the barriers and facilitators to implementing the program. Specifically, findings suggest that internal housing expertise, leadership support, and flexibility show that AAAs can play a role in housing strategies for vulnerable older adults.

## Background and objectives

Population aging has increased the number of older adults in the United States (Vespa et al., 2020), which in turn has increased the demand for services that support healthy aging and prolong community-based living (Yu et al., 2024). This demand has outpaced both the supply of care providers and public investment, making needed services unaffordable for many older Americans (Scheckler et al., 2025). Area Agencies on Aging (AAAs) are mandated by the Older Americans Act to provide and coordinate publicly funded home and community-based services (HCBS) for older adults (Congressional Research Service, 2024, p. 1). There are over 600 AAAs nationwide, although all AAAs share the mandate, there is wide variance in the structure of organization, service, and funding. The HCBS they provide are contingent on older adults in their communities having access to stable and safe ­housing, in which services can be received (Bardo et al., 2014; Jopson & Regan, 2016). Yet, the number of older adults with “worst case housing needs” (i.e., renters, very low income, paying more than 50% of income on rent, do not receive housing assistance) rose in the United States from 1.47 million in 2011 to 2.35 million in 2021 (Alvarez & Steffen, 2023).

The costs of living generally, and specifically the cost of housing, have outpaced the public financial benefits that constitute the “fixed incomes” of many low-income older adult households in the United States (Mutchler & Li, 2020). In 2021, 11.2 million older adult households were housing cost burdened, an increase of 2.4 million since 2011 (Joint Center for Housing Studies, 2023). The gap between retirement income and housing costs is most pronounced for renters, who are more likely than homeowners to live in poverty, have difficulty paying housing costs, and be financially strained (Mehdipanah et al., 2022). Older adults with fixed incomes living in increasingly expensive rental housing often have little residual income and are at risk of losing housing as a result of a singular event (death of a partner, sale of the property) or age-related change (cognitive decline, shrinking social network) (Lee et al., 2016; Scheckler et al., 2023). Living in unaffordable and low-quality housing has been associated with poor self-rated health (Bhat et al., 2022; Jenkins Morales & Robert, 2022; Mehdipanah et al., 2022), chronic conditions (Bhat et al., 2022), functional limitations (Jenkins Morales & Robert, 2022), and moving to a skilled nursing facility (SNF) (Jenkins Morales & Robert, 2020).

The consequences of unaffordable housing undermine the AAAs’ mandate to promote healthy and safe aging (Congressional Research Service, 2024). In response, AAAs have increasingly invested in programs and partnerships to address housing insecurity and homelessness among older adults. According to a 2023 national review of AAA services, 16% of AAAs had a homelessness intervention program, 11% had a homelessness prevention program, 21% offered rental assistance, 3% operated an emergency homeless shelter for older adults, and 71% had partnerships with housing and homeless agencies (USAging, 2023a).

Although AAAs do not all look the same, their federal mandate makes AAAs represent a promising context in which to address housing challenges experienced by older adults. AAA clients tend to be older, lower-income, and living with a disability than the general population of older adults in the United States; characteristics that together pose barriers to residing in affordable and accessible housing (Popkin et al., 2022). AAA services generally have established protocols for routine assessment of clients’ needs and risk factors, and these protocols can generate the timely and relevant insights needed to deliver homeless prevention services efficiently (Shinn et al., 2013). Further, AAAs are community-based organizations that operate outside of the centralized homeless response system, currently referred to as Coordinated Entry (U.S. Department of Housing and Urban Development, 2024a). This positioning may help counter the institutionalization of individuals who are homeless, a consequence of the United States’ centralized and reactive approach (Lindblom, 1991), which often relies on settings (e.g., congregate shelters) and procedures (e.g., waiting lists) that are ill-suited to the needs of aging individuals (Traver et al., 2025).

An emerging housing intervention that may be relevant to housing-insecure older adults with fixed incomes is shallow rental subsidies (Culhane et al., 2020; Henderson et al., 2023; U.S. Interagency Council on Homelessness, 2023). Shallow rental subsidies provide renters at high risk of eviction or homelessness with a modest, recurring level of rental assistance, typically in the absence of “deep” subsidies offered by U.S. Department of Housing and Urban Development (HUD) programs such as Housing Choice Vouchers and Public Housing (HUD, n.d.). In 2023, the San Diego, CA Department of Homeless Solutions and Equitable Communities launched a shallow rental subsidy program (SRSP) that targets renters aged 55 and older whose income was below 50% of the area median and spent more than 50% of their income on housing costs (Saunders, 2023). The program is subject to a randomized evaluation, but no findings have been published to date. In 2024, the Central Ohio Area Agency on Aging (COAAA) launched an SRSP targeting HCBS clients whose incomes were below 50% of the area median and spent more than 50% of their income on their rent. The COAAA program is the focus of this implementation evaluation.

### The implementation context

Central Ohio Area Agency on Aging is one of 12 AAAs in the state of Ohio and serves the central eight-county region, including the state capital, Columbus. Over 80% of COAAA’s funding is from the federal government (Older Americans Act, Medicaid waivers), and about 5% of funding is from city, county, or state governments (COAAA, 2023). COAAA operates three HCBS programs targeted by the SRSP. (1) Senior Options provides HCBS to adults aged 60 and older with at least one unmet care need and is funded through a county-level property tax levy and fees that are charged to clients based on their income and assets. Many clients pay nothing (COAAA, 2024a). (2) PASSPORT is a Medicaid-waiver program that provides HCBS to adults aged 60 and older who earn less than $2,829 per month, have less than $2,000 in countable assets, and are eligible for skilled nursing SNF-level of care (Ohio Department of Aging, 2024). (3) MyCare Ohio is a managed care program that provides HCBS to people enrolled in both Medicaid and Medicare benefits (COAAA, 2024b).

The COAAA SRSP is administered within the agency’s Housing Assistance Program (HAP). Although AAAs are starting to include housing assistance (USAging, 2023a), the HAP is unique to COAAA. Since HAP’s inception in 2019, the program has supported the housing needs of COAAA clients in a variety of ways, including covering past-due rent, paying utility bills, purchasing household goods and services, and completing emergency rental assistance applications. However, the increasingly chronic nature of clients’ housing insecurity motivated the development of a more robust intervention.

### The implemented program

In spring 2024, COAAA launched the SRSP. The SRSP paid $330, the equivalent of 35% of the local fair market rent (HUD, 2024b), directly to the housing providers each month for 12 months. Eligibility criteria included being a current HCBS client with worst-case housing needs, defined as those who rented, did not receive housing assistance, had income less than $36,200 for a single adult, $41,350 for a couple, and paid more than one-half of their income for rent (Alvarez & Steffen, 2023).

### Study aims

The ongoing development of housing and homelessness programs developed by AAAs warrants systematic evaluation to better understand how such programs are being implemented within this specific, well-established service and administrative context. Implementation research serves to enhance the adoption of strategies across service delivery systems and to improve outcomes for clients (Peters et al., 2013). Previous implementation research with AAAs has focused on care transitions (Coe et al., 2023) and an aging-in-place intervention (Toto et al., 2023). Although AAAs are long-standing and robust sites of older adult programming, the body of AAA implementation science is notably small. Through this research, we seek to contribute to this emerging and timely field of study.

To study the SRSP, we employed the Consolidated Framework for Implementation Research (CFIR) (Damschroder et al., 2022). The CFIR was developed to predict or explain barriers and facilitators to implementation effectiveness across five domains: (1) Innovation (the intervention or change being implemented), (2) Outer Setting (the setting in which the inner setting exists), (3) Inner Setting (the setting where the innovation is being implemented), (4) Individuals (the roles and characteristics of the people involved), and (5) Implementation Process (the activities and strategies used to implement the innovation) (Damschroder et al., 2022). Due to the novelty of operating recurring rental assistance in an AAA, this study focused on the inner setting domain, aiming to identify facilitators and barriers related to an AAA’s implementation of a HAP to advance future efforts to aid older adults residing in unaffordable rental housing.

## Research design and methods

The overall evaluation of the SRSP followed an effectiveness-implementation hybrid design (Curran et al., 2012). COAAA aimed to enroll 100 clients in the randomized evaluation. Ultimately, 40 participants were enrolled in the 1-year program beginning in the summer of 2024 due to lower-than-expected referrals during the enrollment window. Twenty were assigned to receive the SRSP, and 20 were assigned to the treatment-as-usual comparison group. This article focuses on the implementation arm of the study.

The implementation arm followed a sequential (QUAN > QUAL) mixed methods (Creswell & Plano Clark, 2017) implementation science design, assessing staff experiences and perceptions of implementation barriers and facilitators across the inner setting. The Inner Setting Domain includes 11 constructs: (1) Structural Characteristics, (2) Relational Connections, (3) Communications, (4) Culture, (5) Tension for Change, (6) Compatibility, (7) Relative Priority, (8) Incentive Systems, (9) Mission Alignment, (10) Available Resources, and (11) Access to Knowledge and Information.

### Recruitment

All staff at COAAA, regardless of role or familiarity with the SRSP, were invited to complete a survey following an all-staff meeting. Members of the research team attended the staff meeting to describe the study and answer questions. Staff who completed the survey were invited to participate in a future focus group. Additionally, all members of the HAP were invited to participate in a separate focus group. Lunch was provided for focus group ­participants.

### Data collection and analysis

Consolidated Framework for Implementation Research developed the pragmatic context assessment tool (pCAT) as a practical method to evaluate the implementation context of a new intervention (Robinson & Damschroder, 2023). We adapted the pCAT to ensure the language was consistent with the AAA setting. For example, “clinical values” was changed to “professional values,” since all staff, not just those in clinical roles, were invited to complete the survey. We also removed the item regarding adequate “space” because COAAA clients are served in their homes and communities. Lastly, we added questions about familiarity with and concern about housing affordability for older adults. An online survey composed of the adapted pCAT (Supplementary Material) was created in Qualtrics, 2024.

Staff were invited to complete the survey at an all-staff meeting in September 2024. The survey was distributed virtually during the meeting, and an emailed link was provided following the meeting. The survey did not force responses to each item, so participants could choose to skip questions. Surveys were anonymous, but participants interested in joining a focus group were asked to provide their email addresses for follow-up. We performed a descriptive analysis of survey responses, including frequency distributions across the pCAT Likert Scales, to identify topics with high disagreement or response variation. This information was used to develop the focus group questions. The focus group questions focused on communication, access to information, and compatibility within the inner setting. The main purpose of the focus groups was to understand how the SRSP fit within current workflows and how staff received information about the program.

Survey participants who expressed interest in joining a focus group were contacted via email. Two general staff focus groups took place on one day, whereas the HAP team focus group was held on a different day. Two research team members attended each focus group, one to facilitate and one to take notes. The sessions lasted 1 hr each and were recorded and transcribed. Due to agency policy, we were unable to provide a research incentive for survey participants; however, focus group participants were provided lunch during the focus group as a nonmonetary incentive for participation. We used a rapid structured approach (McNall & Foster-Fishman, 2007) to analyze the three transcripts. Rapid analysis is a team-based method that involves using a structured summary table with prespecified domains to systematically organize qualitative data, often used in intervention research (Beebe, 2014). The analysis team included four individuals: the two Co-PIs, a PhD candidate, and a Master of Social Work student. This systematic method enables quick sharing of findings with community partners (McNall & Foster-Fishman, 2007), which was important to our team so our partner could use the findings for potential future iterations of the program.

## Results

### Sample characteristics

As described in Table 1, 85 staff members completed the pCAT survey. Just over half (53%) of participants were case managers, and 81% were aware of the SRSP before completing the survey. Although most survey participants were aware of the SRSP, 68% did not make a referral to the program.

**Table 1:** Survey respondent description.

Characteristics	*n* (%)
**Roles**	
**Aging programs care coordinator (case manager)**	45 (53%)
** Aging programs clinical manager**	8 (9%)
** Assessment**	5 (6%)
** Care coordination assistant**	3 (4%)
** Community outreach specialist**	3 (4%)
** Division director**	2 (2%)
** Office assistant**	1 (1%)
** Screening**	1 (1%)
** Other**	15 (18%)
**Referrals to housing assistance program**	
** Have referred**	33 (39%)
** Have not referred**	33 (39%)
** Did not respond**	19 (22%)
**Awareness of shallow rental subsidy program**	
** Aware**	69 (81%)
** Not aware**	16 (19%)
**Referrals to shallow rental subsidy program**	
** No referral made**	58 (68%)
** Referral made and client assigned to SS group**	6 (7%)
**Referral made and client assigned to treatment-as-usual group**	1 (1%)
** Did not respond**	19 (22%)

**Table 2:** Summarizes the inner-setting findings of each construct.

Inner setting construct	Facilitators	Barriers
**Structural characteristics**	Existing structure of the HAP	Limitations to available documentation in existing systems
**Communications**	Formal communication processes	Limited communication about changes to the referral process
**Culture**	The “COAAA Way” centering clients’ needs in service delivery	Concern of adding stress to clients
**Tension for change**	Strong sense that addressing housing was something that COAAA should be doing	
**Compatibility**	Existing structure of the HAP team to address some housing needs HAP staff do not carry a caseload, so they were able to be flexible in screening and enrollment tasks	Misalignment with case manager workflow
**Relative priority**	HAP was instructed by leadership to prioritize the program during the screening and enrollment phases Prioritization was modeled by leadership by joining in screening and enrollment tasks	Large and demanding caseloads for case managers
**Incentive systems**		No incentive program due to public agency regulations
**Mission alignment**	Consensus that the program provided a resource that some clients needed to “live with dignity and independence” Innovative service	
**Available resources**	Funding from the American Rescue Plan Act	Funding limitations capped assistance at 12 months Funding timeline constraints inconsistent with programmatic timelines
**Access to knowledge and information**	Information was shared in several ways	Accelerated timeline forced quick changes that caused confusion

*Note.* COAAA = Central Ohio Area Agency on Aging; HAP = housing assistance program.

Twelve survey participants expressed interest in participating in a focus group. Three focus groups were conducted, two with general staff (*n* = 12) and one with HAP staff (*n* = 5).

### Inner setting domain findings

#### Structural characteristics

Structural characteristics refer to the components of the infrastructure that allow the inner setting to function (Damschroder et al., 2022). Structural characteristics can include components of the physical infrastructure, information technology (IT) infrastructure, and the work infrastructure. The majority of participants reported that IT (82%) and work (83%) infrastructures supported their abilities to implement the SRSP.

However, focus group participants highlighted barriers to structural characteristics. Though 82% of survey respondents agreed that IT supports the functional performance across the agency, focus group participants shared limitations in the electronic documentation available within their existing system, which made checking eligibility difficult. Focus group participants shared that the structures in place did not allow for asking about housing cost burden, which some reported made the SRSP feel like it was outside the scope of their current job. As one participant shared, “Because the clients in my program are Medicaid eligible, they’re…probably eligible for this program, but there’s still the documentation, and we don’t have those numbers in our system. That’s not part of our assessment process.” Focus group participants also shared that the timeline for screening for eligibility did not fit within the structures and cadence required within their respective programs’ home visits with clients.

Focus group participants identified the well-established HAP as an important structural facilitator for implementing the SRSP. The structure of the HAP, including staff familiarity with housing-related resources and their availability to meet potential participants, proved an important aspect of the implementation of the SRSP. The HAP staff had the flexibility to meet with potential shallow rental subsidy recipients because they do not carry a caseload. Additionally, participants in the case manager focus group highlighted the importance of having internal staff who are familiar with housing needs.

### Communications

Communications refers to high-quality formal and informal information-sharing practices within the Inner Setting (Damschroder et al., 2022). Most survey participants (71%) reported that they felt there were high-quality informal and formal processes for sharing information. When asked about the most effective communication method within COAAA for learning about a new program, two avenues were highly ranked: all staff meetings (71%) and hearing about programs directly from ­supervisors (65%).

Focus group participants had varied experiences with how information was shared. Although some focus group participants shared that they did not receive any information other than an announcement at an all-staff meeting, as one participant shared, “I don’t remember getting anything, any information about it, other than maybe it being mentioned in an all-staff.” Others shared that they received information in a variety of ways. Some said they found out about the SRSP through formal processes, including a webinar, written materials, and emails, and directly from leadership. Some shared that they did not hear about the program directly from supervisors, but they felt comfortable going directly to COAAA leadership with questions, as multiple participants shared, “I don’t think I talked to my supervisor about it. I think I went straight to [leadership]” and “I did reach out to [leadership] at the time to ask a few questions. He was able to give me more background information and directions.” Although the multiple forms of communication allowed some case managers to understand the program, it also contributed to confusion about the details of the program. Participants in the focus group with the HAP shared that the referral process changed along the way, which could have contributed to confusion as well, as one participant shared, “We did a recorded webinar to talk about what we would need from [case managers]. Within probably a couple of weeks, that was all different.”

### Culture: recipient-centeredness

Culture refers to the shared values, beliefs, and norms across the inner setting. Specifically, recipient-centeredness refers to the shared values, beliefs, and norms related to addressing the needs and welfare of recipients (Damschroder et al., 2022). Eighty-eight percent of survey participants reported that there were shared values, beliefs, and norms in general across the agency, and 100% of survey participants reported shared values, beliefs, and norms when it comes specifically to supporting the needs and welfare of clients. Similar themes were evident in the focus groups.

Members of the HAP focus group discussed what they called “The COAAA Way”—meeting clients where they are and centering their needs to provide a high level of service. In relation to the SRSP, this meant doing home visits to screen for eligibility, even if that is typically outside one’s normal work responsibilities. These visits included supporting potential clients through the eligibility review with documentation collection, which was an identified barrier for many in enrollment. As another example of the “COAAA Way” and client-centeredness, the HAP team provided an additional touchpoint for clients enrolled in the program. They completed monthly check-ins with program participants to ensure their engagement with the program was running smoothly and landlords were receiving the payments. A member of the HAP team shared that from these monthly check-ins, she built rapport with clients and has become a trusted contact for housing-related questions.

### Tension for change

Tension for change refers to the perception that practices before implementing the new intervention are inadequate and need to change (Damschroder et al., 2022). Staff expressed a high awareness of their clients’ housing challenges. Most survey participants (93%) expressed great concern about affordable housing options for older adults, and 71% reported frequent discussions about affordable housing with clients, colleagues, and/or supervisors. Importantly, 72% of staff shared that they very frequently or often encounter a client struggling to afford housing. Although staff expressed concern and familiarity about housing needs, focus group participants shared that practices prior to the shallow rental subsidy to address housing needs were insufficient.

Focus group participants described addressing client housing needs before the SRSP by making referrals internally to HAP or by providing clients with information about housing services in the community. Although focus group participants shared that working with HAP was straightforward, the emergency rental assistance is not always sufficient because it is one-time assistance, and clients must prove they can sustain their housing after they receive the assistance. They also shared that it often felt as though they lacked the necessary expertise and time to help clients navigate housing resources in the community. Participants talked about the insufficient response of handing a client a list of housing resources, “It’s a huge packet. I’ve had three people this year in housing crisis situations, and I hand them a packet. That’s all I can do within the confines of my job. I want to do more, but I can’t, so I have to find someone else who can do that.” Participants also addressed housing needs by finding creative solutions to leverage services and educate clients about ways to offset non-rent expenses (food, transportation, etc.) to allow more income to go towards rent. As one participant shared, “…there’s aspects of trying to take the financial burden off of the client through offering other supportive services, the idea being that it could free up funding for housing…” In other words, case managers reported that housing was a need that should be addressed, and practices before the SRSP did not directly address the issue.

### Compatibility

Compatibility refers to the intervention being aligned with current workflows, systems, and processes (Damschroder et al., 2022). Staff who completed the survey were asked whether they agreed with the statement, “The SRSP fits within workflows, systems, and processes.” Of the 42 staff who responded to the question, 67% agreed, 26% were neutral, and 7% disagreed.

Focus group participants shared several barriers to compatibility with workflow and systems. Focus group participants shared that acquiring documents, such as a lease and proof of income, did not fit into the case manager workflow, that there was not enough time, or that the timing was not aligned with their visit schedule. As one focus group participant shared:

I think it was around a month time period we had to get the information and stuff as far as whom we wanted to refer. For our program, we see people every three months, so it was trying to remember who off your caseload had mentioned being rental burdened or financially challenged as far as housing, and then reaching out to them extra above what your normal duties were, which–doesn’t leave much leeway to be able to do that.

Additionally, elements of the program were outside the scope of work for some, whether that be conducting home visits for the HAP team or collecting rent and income information for the case managers.

Focus group participants shared two primary facilitators that enhanced the compatibility of the new program. First, the ability and willingness of those in leadership positions to step in and actively help with the screening and enrollment process. Second, the flexibility of the HAP team to step outside of their typical role and assist case managers by going to clients to gather paperwork. As one participant in the HAP focus group shared, “Almost all of us went out and gathered that documentation ourselves if the case manager wasn’t available to do that. There was a lot of going out to homes, which was unusual for us in the housing department.”

### Relative priority

Relative priority refers to the perception that implementing the new intervention is important compared to other initiatives (Damschroder et al., 2022). Survey participants were asked if they agreed or disagreed that implementing the SRSP was important compared to other COAAA initiatives. Seventy-five percent of the 52 who answered the question agreed with the statement; 21% were neutral, and just 4% disagreed. Although most survey participants reported that implementing the SRSP was important, case managers who participated in focus groups shared that it was difficult to prioritize the SRSP with the other priorities of their daily work. For example, one participant shared, “…navigating your regular case manager duties among all the other resources that are available. By the time I did reach out, it was past, or it was already full.” Others felt that implementing the project by referring and screening clients was outside their scope of work, both in practice and in expertise.

The HAP team shared that the case managers’ heavy workload contributed to a slow start of referrals and led to a shift in the screening process. As one focus group participant shared:

People were confused because we had initially decided that we were going to get a lot of information on the frontend from the case managers…what we found out pretty quickly, because of the lack of referrals coming in, was that was too much information to put on the case managers to essentially do the screening. That’s fine, they have their own jobs.

Because the program was seen as a high priority for COAAA leadership and the HAP team, the HAP team and leadership engaged with clients for eligibility and enrollment rather than the case managers. Essentially, the leadership instructed the HAP team to prioritize the SRSP, which was modeled by the leadership team as they were also reaching out to clients and screening for eligibility.

### Incentive systems

Incentive systems refer to the tangible or intangible incentives and rewards or disincentives and punishments that support the implementation and delivery of the program (Damschroder et al., 2022). Just 43% of the survey respondents agreed that the agency provided incentives for implementing the program. One consideration that complicates the potential incentive systems is that policy limits employees’ ability to receive monetary incentives for work activities. During all three focus groups, facilitators included a question related to incentives, and there was nothing of note shared.

### Mission alignment

Mission alignment suggests that implementing and delivering the new intervention aligns with the mission and goals of the inner setting (Damschroder et al., 2022). COAAA’s mission and vision are as follows: “The mission of the Central Ohio Area Agency on Aging is to inform and support people as they navigate the experience of aging or disability. Our vision is for individuals and families to have knowledge and access to the information and resources they need to live life with dignity and independence” (COAAA, n.d.). Most staff who completed the survey (88%) agreed that the SRSP aligned with the mission and goals of COAAA, 12% were neutral, and zero respondents disagreed. Focus group participants shared that the program aligned with COAAA’s mission to be innovative with service delivery. As one participant shared, “…one of the reasons why I was really excited when I was interviewing was because COAAA is doing things like this. Yes, we have to be careful. At the same time, the fact that we are trying these initiatives…the benefits outweigh the risk.”

### Available resources

The construct of available resources refers to the availability of resources needed to implement and deliver the innovation. This can include funding, physical space, and materials and equipment. The survey included one question about program funding. Forty survey participants answered this question, and of the 40 respondents, 56% felt that the program was adequately funded. The majority of program funding came from the American Rescue Plan Act (ARPA), which influenced how and when the program was implemented. The stipulation that funds be spent by a certain date and the desire to provide the shallow rental subsidy for 12 months created a sense of urgency and a time program during the implementation.

### Access to knowledge and information

Access to knowledge refers to the guidance or training that is available to implement the new program (Damschroder et al., 2022). Forty survey participants answered this question, and of those 40, 42% felt they did not receive guidance or training to implement the SRSP. Focus group participants reported similar barriers to accessing information about the program. Many reported not understanding the eligibility criteria and enrollment procedures, while a few were unaware of basic program features, such as the recurring payments, the program’s length, and the impact of the payments on clients’ benefits. Focus group participants shared that they learned about the program when it was announced at an all-staff meeting that was followed by an email and a webinar. However, according to a member of the HAP, the short timeline led to quick changes that could have contributed to confusion about the program, sharing, “We did do a recorded webinar to talk about what we would need from them (case managers). Within probably a couple of weeks, that was all different.”

The confusion about elements of the program and the evaluation component did influence whether and how case managers told clients about the SRSP. One participant shared that she wanted more information about the rationale behind it being a one-year program, stating, “One year. My concern is what was being communicated to people? Because that can be retraumatizing for somebody if they’re getting a subsidy for a year and then all of a sudden they can’t afford it.” Similar to the concerns about the misalignment between the evaluation and the client-centered culture of the agency, case managers expressed a desire to know more about the program so they could effectively address questions and concerns from clients.

## Discussion and implications

As the U.S. population ages, older adults are navigating aging with less savings, more debt, and higher housing costs than previous generations (Joint Center for Housing Studies, 2023). This reality challenges the work of AAAs, who are federally mandated to promote independent and safe aging for all older adults in the United States (Congressional Research Service, 2024). Unaffordable housing undermines this mandate, and in response, AAAs are investing more resources into programs and partnerships focused on housing insecurity and homelessness (USAging, 2023a). As AAAs navigate this new challenge, there is little evidence on the best ways to implement these new programs and partnerships. Our mixed methods study, guided by the CFIR framework (Damschroder et al., 2022), identified facilitators and barriers to implementing a housing program in the AAA setting. Below, we summarize these facilitators and barriers and discuss opportunities for program reform that can guide future housing initiatives across the AAA network. Although the barriers and opportunities are specific to the context at COAAA, there is a broad application to the AAA network given their shared mandate and challenges.

### Facilitators

One of the most prevailing facilitators was the existence of the HAP and its staffing structure. Having housing experts on staff, particularly as a team, allowed for flexibility to create and implement the new housing program. Although many AAAs are establishing programs or partnerships around housing (USAging, 2023b), COAAA is unique in the longevity and scale of its existing infrastructure to address clients’ housing needs. Additionally, while most AAAs address client housing needs by partnering with community housing and homelessness agencies (USAging, 2023a), the HAP and SRSP are unique in that they are housing interventions that were implemented within an AAA. The HAP as an internal entity was an integral part of implementing the SRSP, both for their understanding of housing resources and for their flexibility to screen potential participants for eligibility and follow up with participants once enrolled. It is also critical to understand how best to integrate housing-focused staff within AAAs. Future research should assess the interactions and processes between program case managers and housing staff, such as those in COAAA’s HAP team, to optimize workflow between the two roles. Our findings regarding program implementation facilitators provide important insights for other AAAs on the value of building internal structures and investing in staff who can support existing programs that address housing issues.

Eighty-five percent of AAAs consider housing affordability a major challenge (USAging, 2023a), indicating an opportunity for similar programs that prioritize housing across the AAA network. Assessing housing challenges early and often is critical for homeless prevention (Nicholas & Henwood, 2018; Shinn et al., 2013), and may represent one way that AAAs can counter the rise of older adult homelessness in the United States (Byrne et al., 2024; Culhane et al., 2019). Staff in AAAs around the country are in the homes of millions of older adults, meeting them where they are to address their needs and allow them to age in the community. COAAA is no different. The only survey metric that received unanimous agreement was the existence of shared values, beliefs, and norms around caring for and supporting clients’ needs and welfare. The culture at COAAA of centering clients’ needs played an important role in implementing the SRSP. The importance of centering clients’ needs can be translated across the AAA network. Unlike many traditional homelessness prevention or housing support resources, where the onus is on the client to gather all documentation to access housing resources, COAAA staff met clients in their homes and helped them gather the necessary documentation. Although it became clear that this approach was an unreasonable expectation of case managers, the existing culture led to an “all hands on deck” mentality where leadership and HAP staff were meeting with clients for screening and enrollment. The program was also a high priority for HAP staff and COAAA leadership, which was evident in how they completed tasks that would typically be outside of their roles.

### Barriers

Predominant barriers to implementing the SRSP included caseload and time constraints of case managers, document acquisition, and confusion around the program and eligibility criteria. These barriers contributed to a lower-than-expected number of referrals from case managers, a key component of the implementation process. COAAA initially planned to enroll 100 participants in the SRSP, but ultimately enrolled 40 participants due to low referrals and a short enrollment window. Although the flexibility of HAP staff allowed the program to continue, AAAs should consider ways to allow more case manager involvement in the referral and screening process when implementing the program at scale. Potential solutions are rolling enrollments and consistent data collection to facilitate faster referrals to housing-related services.

It is important to consider how funding timeline constraints contributed to implementation barriers. The urgency related to the funding timeline resulted in a relatively short screening and enrollment period, which rushed some of the implementation procedures, potentially negatively affecting other constructs such as communication, compatibility, and access to knowledge and information. Although this analysis intentionally focused on the inner setting domain, additional research that includes the other CIFR domains may provide additional insights into barriers from the funding timeline constraints.

Focus group data revealed that front-line staff had a limited understanding of the SRSP, including who the program targeted and what it provided. When the program was understood, its implementation was considered by case managers to be beyond their scope of practice or an additional burden that their workload could not accommodate. Acquiring the documentation for eligibility screening and enrollment often meant home visits that were off-schedule and, therefore, not feasible to fit into their schedules. Further, many staff expressed that they did not feel confident dealing with the complexity of clients’ housing challenges due to a lack of housing-specific expertise. Clients of COAAA, given the eligibility criteria and requirements, often present with deeply complex and layered challenges for case managers to navigate. Additionally, aging services continue to be a sector with significant workforce shortages. These two factors are often noted by case managers related to burden, something also cited in relation to understanding and referring to a new program.

### Opportunities

Results of this implementation study identify opportunities across multiple constructs to enhance the implementation of future rental assistance programs in AAAs. For example, a more targeted approach to communication or training with each department around the purpose of the evaluation, details of the program itself, and talking points could help ease concerns about the SRSP evaluation. When asked about preferred communication methods to learn about new programs within COAAA, survey participants shared that all-staff meetings and hearing directly from supervisors were the most effective methods. Although survey and focus group participants shared that they did hear about the program at an all-staff meeting, they did not hear about it directly from supervisors, so prioritizing communication directly from supervisors is an opportunity to improve program implementation.

One clear opportunity is within the incentive systems domain. COAAA did not attempt to incentivize implementation activities due to agency policies that prevent the provision of money, gifts, or special benefits to employees for activities completed in their employment capacity. This challenge will likely be encountered by other AAAs that are public agencies or have unionized staff. Identifying and providing permissible incentives to staff will be an important step for future implementation, which would need to comply with agency-specific strategies. Future studies should collaborate with staff to figure out what kinds of incentives would be of value when implementing the program.

Another opportunity relates to the construct of structural characteristics, specifically the maintenance and accessibility of client data on housing insecurity. Previous research has highlighted the role of information management in service quality and equity for vulnerable populations (Rajaram et al., 2020), and this was a domain pointed to by focus group participants. In order to mitigate the issue of access to housing-related data, focus group participants proposed routine and systematic collection of housing characteristics, such as rental costs, eviction notices, or maintenance requests. Such information, if stored in an accessible platform, would provide a more accurate understanding of clients’ needs and risks while streamlining the referral process. Of course, gathering such information requires additional time and energy, which was generally considered to be in short supply. Interestingly, agency staff stated that such routine data collection would not be burdensome if incorporated into an assessment or routine visit, despite previously stating that collecting data for program eligibility was too burdensome and beyond their scope of practice. Such a discrepancy provides additional evidence of staff perceptions of the importance of understanding clients’ housing situations and suggests that routine data collection is potentially the best way to incorporate a new task into current practices. It also reflects a need for implementation research specifically exploring how AAAs implement new housing insecurity assessment protocols. Such research would help answer important questions about how, when, and by whom such information is best collected and utilized, and contribute to the growth of implementation science within AAAs.

### Limitations

As with all studies, this study is not without limitations. First, this implementation assessment focused solely on the inner-setting domain. Although this decision was made to understand the implementation of a program within the unique context of an AAA, the current rapid changes in larger systems and funding mechanisms highlight the importance of understanding how the other CFIR domains may have affected the SRSP implementation.

The funding mechanism required a shortened referral, screening, and enrollment process, which appears to have influenced the implementation of the program. The findings have limited generalizability because of the unique funding available at the specific time.

There is potential for selection bias for both the surveys and, in particular, the focus groups. Additionally, although 85 COAAA staff completed the survey, many skipped questions. In particular, the questions about incentives, funding, and adequate training had low response rates. This could be due to confusion about the questions or the constructs themselves. Although the surveys were anonymous, staff completed the surveys at their place of work, which may have influenced the way they answered (or did not answer) questions.

### Conclusion

Area Agencies on Aging are uniquely positioned to address the rising rates of housing insecurity among older adults in the United States. Unlike many organizations within the housing and homelessness system, AAA staff generally meet clients in their homes and, therefore, directly see the impact of the housing affordability crisis. Meeting clients in their homes gives AAA staff the unique opportunity to closely support a client navigating the often burdensome processes and requirements to access housing support. Although our study focused on the inner setting CFIR domain, implementing novel housing programs such as the SRSP is largely contingent on funding. Improving the implementation of shallow rental subsidies within AAAs is dependent on funding such programs and the continued federal funding of AAAs through the Older Americans Act. As AAAs around the country begin to have more housing programs and partnerships, understanding implementation within their unique context is critical for success. The focus group participants in this study expressed a strong desire for more options for long-term housing supports in addition to the one-time resources that were in place at COAAA. AAAs can build from this tension for change to implement targeted housing resources, such as shallow subsidies, to better meet the needs of the individuals they serve.

## Supplementary Material

igag015_Supplementary_Data

## Data Availability

Data are available from the corresponding author upon reasonable request, subject to ethical approval. This study was not preregistered.
