# Supplementary material for: Barriers and facilitators of implementing a shallow rental subsidy program at an area agency on aging
Source: Innov Aging. 2026 Feb 13;10(5):igag015. doi: 10.1093/geroni/igag015 (PMC13069934; doi:10.1093/geroni/igag015)
Supplement: igag015_Supplementary_Data [file igag015_supplementary_data.pdf]

Innovation in Aging **Supplementary Material: Calhoun, Traver, & Sheldon. (2026). Barriers and facilitators of implementing a shallow rental subsidy program at an Area Agency on Aging.**

COAAA Shallow Subsidy Inner Setting Survey

Thank you for participating in this survey on affordable housing for older adults and the implementation of COAAA's Shallow Rental Subsidy Program. Your insights are invaluable in helping us understand the program's impact and identify areas for improvement.

Instructions:

1. **Purpose:** This survey aims to gather your feedback and experiences regarding your perception of housing stability for older adults and the implementation of the Shallow Rental Subsidy Program. Your responses will be used to assess the effectiveness of the program and to guide future decisions.
2. **Anonymity:** Your responses are completely anonymous. Please answer each question as honestly and accurately as possible. There are no right or wrong answers, and your candid feedback is greatly appreciated.
3. **Survey Structure:** The survey consists of multiple-choice questions, Likert scale questions, and a few open-ended questions. The survey should take approximately 10-15 minutes to complete.
4. **Completeness:** Please ensure you answer all the questions. If you encounter any questions that you feel do not apply to you, please select the "Not Applicable" or "I don't know" option if available.
5. **Submission:** Once you have completed the survey, please click the "Submit" button at the end of the questionnaire. You will receive a confirmation message once your responses have been successfully submitted.
6. **Assistance:** If you have any questions or need assistance while completing the survey, please contact Katie Calhoun at calhoun.284@osu.edu or 614-292-3367.

Thank you again for your time and valuable input. Your participation will contribute significantly to the success of the Shallow Rental Subsidy Program.

1. What is your role at COAAA?
- Aging Programs Care Coordinator (case manager), Aging Programs Clinical Manage, Division Director, Care Coordination Assistant, Housing-Community Relations Coordinator, Screening, Assessment, Community Outreach Specialist, Office Assistant, Other

2. In what COAAA program do you work?
-PASSPORT, MyCare (Aetna/Molina), Senior Options, Other, Not Applicable

3. How concerned are you about the affordability of housing options for older adults in Central Ohio?
-Not at all concerned, slightly concerned, moderately concerned, very concerned, extremely concerned

4. How often do you discuss concerns about housing affordability for older adults with clients, colleagues, supervisors, etc.?
-never, rarely, occasionally, often, very frequently

5. How often do you encounter clients who struggle to afford their current housing?
-never, rarely, occasionally, often, very frequently, I don't work directly with clients

6. Were you aware that COAAA was implementing a shallow subsidy program?
-yes
-no →end survey?

7. Did you screen any clients for program eligibility?
-yes
-no → In a sentence or two, please explain why not. → skip to 8

8. Have you referred any clients to the COAAA housing assistance program?
-yes
-no → In a sentence or two, please explain why not. → skip to 8

9. Were any of your referrals enrolled in the shallow subsidy program?
-yes, subsidy group (receiving the subsidy)
-yes, comparison group (not receiving the subsidy)
-no

--New Section--

The following questions ask about the implementation of the shallow subsidy program at COAAA. The questions refer to practices or attitudes that can be considered facilitators to implementing a new program. You will first answer whether or not you agree that the practice was present for the initial implementation of the Shallow Rental Subsidy Pilot Program. Then, you will be asked to rate how the presence or absence of the practice, resource, or attitude impacted your ability to implement the Pilot Program.

[A. structural characteristics: IT]
Technological systems (communication, documentation, data management) support functional performance across the agency.

1 – Disagree   2 – Neutral   3 – Agree   4-IDK

if 1   “What is the likely impact of the absence of this resouce on your ability to implement the shallow subsidy program?”

0 – weak/no effect   1 – Strong effect

if 2   Skip

if 3   “What is the likely impact of the presence of this resource on your ability to implement the shallow subsidy program?”

0 – weak/no effect   1 – Strong effect

if 4   Skip

[A. structural characteristics: work infrastructure]
Organization and management of staff and teams support functional performance across the agency.

1 – Disagree   2 – Neutral   3 – Agree   4-IDK

if 1   “What is the likely impact of the absence of this practice on your ability to implement the shallow subsidy program?”

0 – weak/no effect   1 – Strong effect

if 2   Skip

if 3   “What is the likely impact of the presence of this practice on your ability to implement the shallow subsidy program?”

0 – weak/no effect   1 – Strong effect

if 4   Skip

[B. relational connections]
There are high-quality formal and informal relationships, networks, and teams within the agency.

1 – Disagree   2 – Neutral   3 – Agree   4-IDK

if 1   “What is the likely impact of the absence of this practice on your ability to implement the shallow subsidy program?”

0 – weak/no effect   1 – Strong effect

if 2   Skip

if 3   “What is the likely impact of the presence of this practice on your ability to implement the shallow subsidy program?”

0 – weak/no effect   1 – Strong effect

if 4   Skip

[C. Communications]

There are high quality formal and informal information sharing practices within the agency.

1 – Disagree 2 – Neutral 3 – Agree 4-IDK

if 1 "What is the likely impact of the absence of this practice on your ability to implement the shallow subsidy program?"

0 – weak/no effect 1 – Strong effect

if 2 Skip

if 3 "What is the likely impact of the presence of this practice on your ability to implement the shallow subsidy program?"

0 – weak/no effect 1 – Strong effect

if 4 Skip

[D. Culture]

There are shared values, beliefs, and norms across the agency.

1 – Disagree 2 – Neutral 3 – Agree 4-IDK

if 1 "What is the likely impact of the absence of such systems on your ability to implement the shallow subsidy program?"

0 – weak/no effect 1 – Strong effect

if 2 Skip

if 3 "What is the likely impact of the presence of such systems on your ability to implement the shallow subsidy program?"

0 – weak/no effect 1 – Strong effect

if 4 Skip

[D. Culture: Recipient-Centeredness]

There are shared values, beliefs, and norms around caring, supporting, and addressing the needs and welfare of clients.

1 – Disagree 2 – Neutral 3 – Agree 4-IDK

if 1 "What is the likely impact of the absence of this practice on your ability to implement the shallow subsidy program?"

0 – weak/no effect 1 – Strong effect

if 2 Skip

if 3 "What is the likely impact of the presence of this practice on your ability to implement the shallow subsidy program?"

0 – weak/no effect 1 – Strong effect

if 4 skip

[E. Tension for change]
Regarding housing support services at COAAA, the current situation is inadequate and needs change.

1 – Disagree 2 – Neutral 3 – Agree 4-IDK

if 1 "What is the likely impact of the current situation of housing support on your ability to implement the shallow subsidy program?"

0 – weak/no effect 1 – Strong effect

if 2 skip

if 3 "What is the likely impact of the current situation of housing support on your ability to implement the shallow subsidy program?"

0 – weak/no effect 1 – Strong effect

if 4 skip

[F. Compatibility]
The shallow subsidy program fits with workflows, systems, and processes.

1 – Disagree 2 – Neutral 3 – Agree 4-IDK

if 1 "What is the likely impact of the absence of this fit on your ability to implement the shallow subsidy program?"

0 – weak/no effect 1 – Strong effect

if 2 skip

if 3 "What is the likely impact of the presence of this fit on your ability to implement the shallow subsidy program?"

0 – weak/no effect 1 – Strong effect

if 4 skip

[G. Relative priority]
Implementing and delivering the shallow subsidy program is important compared to other initiatives.

1 – Disagree 2 – Neutral 3 – Agree 4-IDK

if 1 "What is the likely impact of the absence of this attitude on your ability to implement the shallow subsidy program?"

0 – weak/no effect 1 – Strong effect

if 2 skip

if 3 "What is the likely impact of the presence of this attitude on your ability to implement the shallow subsidy program?"

0 – weak/no effect 1 – Strong effect

if 4 skip

[H. Incentive Systems]
COAAA provided incentives or disincentives to support implementation and delivery of the shallow subsidy program.

1 – Disagree 2 – Neutral 3 – Agree 4-IDK

if 1 "What is the likely impact of the absence of this practice on your ability to implement the shallow subsidy program?"
0 – weak/no effect 1 – Strong effect
if 2 skip
if 3 "What is the likely impact of the presence of this practice on your ability to implement the shallow subsidy program?"
0 – weak/no effect 1 – Strong effect
if 4 skip

[I. Mission Alignment]
Implementing and delivering the shallow subsidy aligns with the overarching commitment, purpose, or goals of the agency.

1 – Disagree 2 – Neutral 3 – Agree 4-IDK

if 1 "What is the likely impact of the absence of this situation on your ability to implement the shallow subsidy program?"
0 – weak/no effect 1 – Strong effect
if 2 skip
if 3 "What is the likely impact of the presence of this situation on your ability to implement the shallow subsidy program?"
0 – weak/no effect 1 – Strong effect
if 4 skip

[J. Available Resources: Funding]
Funding is available to implement and deliver the shallow subsidy.

1 – Disagree 2 – Neutral 3 – Agree 4-IDK

if 1 "What is the likely impact of the absence of funding on your ability to implement the shallow subsidy program?"
0 – weak/no effect 1 – Strong effect
if 2 skip
if 3 "What is the likely impact of the presence of funding on your ability to implement the shallow subsidy program?"
0 – weak/no effect 1 – Strong effect
if 4 skip

[J. Available Resources: knowledge & information]

Guidance and/or training is accessible to implement and deliver the shallow subsidy program.

1 – Disagree 2 – Neutral 3 – Agree 4-IDK

if 1 "What is the likely impact of the absence of this practice on your ability to implement the shallow subsidy program?"

0 – weak/no effect 1 – Strong effect

if 2 skip

if 3 "What is the likely impact of the presence of this practice on your ability to implement the shallow subsidy program?"

0 – weak/no effect 1 – Strong effect

if 4 skip

Thank you for completing the survey. We will be conducting focus groups during a catered lunch at the end of October. Are you interested in participating in a focus group?

Yes No

If no – End survey

If yes – Enter Email address

Questions - Email: Calhoun.284@osu.edu; 614-292-3367
